# P-1986. COVID-19 Surveillance in Infants, Minnesota, October 2023–January 2024

**DOI:** 10.1093/ofid/ofae631.2144

**Published:** 2025-01-29

**Authors:** Abbey J Ruhland, Stephanie Meyer, Kathryn Como-Sabetti, Ashley Fell, Elle Talsma, Siobhan O’Toole, Ruth Lynfield

**Affiliations:** Minnesota Department of Health, St. Paul, Minnesota; Minnesota Department of Health, St. Paul, Minnesota; Minnesota Department of Health, St. Paul, Minnesota; Minnesota Department of Health, St. Paul, Minnesota; Minnesota Department of Health, St. Paul, Minnesota; Minnesota Department of Health, St. Paul, Minnesota; Minnesota Department of Health, St. Paul, Minnesota

## Abstract

**Background:**

The impact of COVID-19 in infants is incompletely understood. We conducted active surveillance, including interviews of parents of infants with COVID-19.

**Methods:**

We attempted phone interviews of parents of infants aged < 12 months with lab-confirmed COVID-19 reported to the Minnesota Department of Health from 10/1/2023–1/31/2024 using a standardized form. Analyses were performed using SAS 9.4.

**Results:**

Parents of 1161 (35%) infants were interviewed. There were no differences among those interviewed vs. not by white/non-white, urban/rural residence, or age. Median age was 6 months (IQR 3–8 months), 65% white, non-Hispanic, 53% male, 46% received federal assistance (WIC), and 38% attended childcare. 25% had an ill household member prior to, and 55% after, infant illness.

95% reported symptoms with fever (84%) and coryza (82%) most common. Co-pathogen detections were reported in 16% (68% RSV).

70% reported emergency (ED) or urgent care (UC) visits; 8% were hospitalized. Having a codetection (OR=4.14, p< 0.01) or underlying condition (OR=2.12, p=0.03) was associated with hospitalization.

16% of illnesses were considered severe by parents. Codetections (OR=2.46, p< 0.01), being non-white (OR=2.46, p< 0.01), receiving WIC (OR=1.99, p< 0.01), or having household income < $30,000 (OR=2.28, p< 0.01) were associated with severe illness. 80% of households had ≥ 1 adult vaccinated, but only 29% were vaccinated < 6 months prior to infant infection; 34% of households with other children had ≥ 1 child vaccinated.

10% of cases remained unrecovered at 30 days after positive test. Factors linked to non-recovery included codetections (OR=2.30, p< 0.01), prematurity (OR=2.19, p< 0.01), being non-white (OR=1.62, p=0.02), receiving WIC (OR=1.56, p=0.03), and low household income (OR=2.09, p< 0.01).

**Conclusion:**

Among MN infants reported with COVID-19 with interview data, most visited ED/UC, and 16% were considered severely ill by parents. We found medical conditions, race, socioeconomic disparities, and codetections associated with severe and/or longer lasting illness. Improved understanding of the role of codetections and other risk factors, and encouraging up to date vaccination in households, may help mitigate the impact of severe COVID-19 in infants.

**Disclosures:**

All Authors: No reported disclosures

